# A Novel Splicesite Mutation in the *EDAR* Gene Causes Severe Autosomal Recessive Hypohydrotic (Anhidrotic) Ectodermal Dysplasia in an Iranian Family

**Published:** 2016-10-23

**Authors:** Shahram Torkamandi, Milad Gholami, Javad Mohammadi-asl, Somaye Rezaie, Mohammad Ali Zaimy, Mir Davood Omrani

**Affiliations:** 1 *Department of Medical Genetics, Faculty of Medicine, Shahid Beheshti University of Medical Sciences, Tehran, Iran.*; 2 *Noor Genetics Laboratory, Ahvaz, Iran.*; 3 *Ahvaz Jundishapour University of Medical Sciences, Ahvaz, Iran.*; 4 *Department of Neurology, Imam Hossein Hospital, Shahid Beheshti University of Medical Sciences, Tehran, Iran.*; 5 *Department of Medical Genetics, Faculty of Medicine, Tehran University of Medical Sciences, Tehran, Iran.*

**Keywords:** Hypohidrotic ectodermal dysplasia, splice site mutation, *EDAR*, c.730-2 A&gt;G

## Abstract

Hypohidrotic ectodermal dysplasia (HED) is a rare congenital disorder arising from deficient development of ectoderm-derived structures including skin, nails, glands and teeth. The phenotype of HED is associated with mutation in *EDA, EDAR, EDARADD* and *NEMO *genes, all of them disruptingNF-κB signaling cascade necessary for initiation, formation and differentiation in the embryo and adult. Here we describe a novel acceptor splice site mutation c.730-2 A>G(IVS 8-2 A>G) in *EDAR* gene in homozygous form in all affected members of a family,and in heterozygous form in carriers. Bioinformatics analysis showed that this mutation can create a new broken splicing site and lead to aberrant splicing.

Hypohidrotic ectodermal dysplasia (HED), the most frequent forms of ectodermal dysplasia is a rare heritable and congenital disorder that results in the abnormal development of ectodermal structures. HED is characterized by a triad of clinical features including hair (hypotrichosis), teeth (hypodontia, anodontia) and sweet gland (hypohidrosis, anhidrosis), the latter of which may potentially lead to life-threatening and brain damage hyperthermia. Other clinical dysmorphic symptoms associated with HED such as peculiar facial features, prominent lip and forehead bumps were reported in some cases (1, 2). Also, dryness of the skin, eye, airway and mucosal membrane which result from exocrine gland dysfunction, leading to eye problems (e.g. chronic conjunctivitis, blepha-ritis), upper respiratory tract infection and atrophic rhinitis, and rarely death have been reported(3). The indistinguishable phenotype of HED is associated with mutation in the encoded X-linked genes ectodysplasin A (*EDA)* andNFκB essential modulator(*NEMO*) or autosomal genes including ectodysplasin A receptor (*EDAR)* and EDAR associated-death domain (*EDARADD)*, all of them disturbing NF- κB signaling cascade necessary for initiation, formation and differentiation of the skin and its appendage. HED is commonly inherited as X-linked form, but autosomal recessive and autosomal dominant conditions are much less common (4). In this study, we performed clinical and genetic investigation on an Iranian family containing seven patients with apparently autosomal recessive form of HED and clinical manifestations including hypotrichosis, anodontia and anhidrosis with a novel mutation at *EDAR* gene.

## Case report

**Fig. 1:**
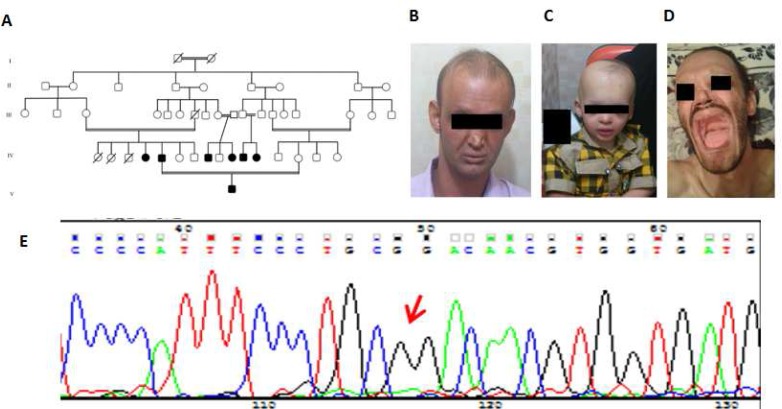
Pedigree, clinical and molecular investigation of ahypohydrotic ectodermal dysplasia (HED) family. (a) Pedigree of an Iranian HED family in this study, IV5 was proband. (b,c,d) clinical feature of patients: sparse hair, eyelashes and eyebrows, anodontia, saddle nose and thick everted lips. (e) The homozygous mutation detected in the *EDAR* gene of affected patients.

The proband (IV 5) was a 30-year-old man born to a consanguineous Iranian family (the second affected of first cousin parents)with HED. His mother and dead father were both clinically normal with no presenting symptoms.Pedigree analysis showed high rate of consanguineous marriage with 7 affected members (4 males and 3 females) in 2 successive generations of the family, indicating that the disease may have an autosomal recessive inheritance pattern in this family (Figure 1). The proband had an affected 2-year-old boy with congenital symptoms of HED. The patient was born at full term of a perfectly normal pregnancy and delivery. His appearance displayed the classical characteristic features of hypohidrotic ectodermal dysplasia: Thin and sparse dry hair over the scalp, scanty eyebrows and eyelashes, dry and finely wrinkled skin, reduced number of eccrine glands, frontal bossing, saddle nose, thick everted lips and sweating had not be observed in response to heat. Dental examination of proband and affected participants, revealed anodontia and absence of any deciduous and permanent dentition.

Blood samples were taken from patients and their non-affected siblings and parents.Genomic DNA was isolated from peripheral blood leukocytes of all available family members (III3, III9, III10, IV5, IV8, IV9, IV10, IV14, V1) using salting out method. All coding exons and the intron/exon boundaries of *EDAR* and *EDARADD *genes were amplified by polymerase chain reaction using gene specific primers. Amplified products were directly sequenced using the ABI Big Dye terminator cycle sequencing kit (Applied Biosystems, Foster City, CA, USA) according to the description of the manufacturer's instruction. Sequences were determined using an ABI 3100x1 Genetic Analyzer (Applied Biosystems). Primer sequences are available upon request.

The only non-polymorphic *EDAR* gene change c.730-2 A>G (IVS 8-2 A>G) was found in homozygous form in patients and heterozygous form in healthy carrier parents and their siblings. The sequencing results of participants was shown in Table1. In order to evaluate the possible impact of IVS8-2 A>G, a novel splice-donor site mutation on *EDAR* transcript splicing, three widely used bioinformatics algorithms for splicing were used: EIEs, ESE-Finder and RESCUE ESE Hexamers (5). We found that this mutation created a new broken exon splicing enhancer (ESE) site located 4 nucleotide downstream of the wild acceptor site. This novel acceptor splice site mutation may therefore have the potential to alter the splicing of *EDAR *transcript, leading to exon skipping and aberrant splicing.

## Discussion

The *EDAR* gene is located on chromosome 2q11–q13 and contains 12 exons. *EADR* encodes a protein which is a member of the NF-κB signaling pathway (5). *EDAR* is expressed during early development and has a crucial role in the development of ectoderm derived structures such as the hair, teeth, glands, scales and nails (6). Here we report a novel, homozygous mutation c.730-2 A>G (IVS 8-2 A>G) in *EDAR*ina large pedigree with high rate of consanguineous marriage and presenting HED. According to pedigree and molecular genetic analyses, the disease has an autosomal recessive inheritance. So in this large family for example III9 and III10 couple have 25% risk of having affected offspring. Pseudo-dominant inheritance can be seen as well for IV5 and IV14 couple with each of their offspring having 50 percent risk of being affected. According to several splice site prediction software analyses, we expect the mutation to cause altered acceptor splicing site in *EDAR* transcript. These classical symptoms of HED including sparse hair over the scalp, scanty eyebrows and eyelashes, wrinkled skin, frontal bossing, saddle nose, thick everted lips and anhidrotic can be in concordance with severe pathogenic effect of this recessive splicing site mutation.

**Table1:** Participantssequencing results

Patients Name	Sex	Age	Relationship	Test	Result
V1	M	2		Sequencing	c.[730-2A>G]; [730-2A>G]
IV5	M	30	Affected father of V1	Sequencing	c.[730-2A>G]; [730-2A>G]
IV14	F	23	Unaffected mother of V1	Sequencing	c.[730-2A>G];[wt]
IV8	M	30	Affected uncle of V1	Sequencing	c.[730-2A>G]; [730-2A>G]
IV9	M	28	Unaffected uncle V1	Sequencing	c.[730-2A>G];[wt]
IV10	F	26	Affected aunt of V1	Sequencing	c.[730-2A>G]; [730-2A>G]
III3	F	66	Unaffected grandmother of V1	Sequencing	c.[730-2A>G];[wt]
III9	F	60	Unaffected grandaunt of V1	Sequencing	c.[730-2A>G];[wt]
III10	M	64	Unaffected granduncle of V1	Sequencing	c.[730-2A>G];[wt]

Taken together, the identified novel homozygous single nucleotide transition in *EDAR *is most likely to explain the clinical symptoms of the patient including: sparse hair, eyelashes and eyebrows, anodontia, saddle nose and thick everted lip sand which lead to severe formof the disease.
